# Quantum Image Processing Algorithm Using Line Detection Mask Based on NEQR

**DOI:** 10.3390/e25050738

**Published:** 2023-04-29

**Authors:** Tao Li, Pengpeng Zhao, Yadong Zhou, Yidai Zhang

**Affiliations:** 1 College of Science, Northwest A&F University, Yangling 712100, China; 2 College of Optical and Electronic Technology, China Jiliang University, Hangzhou 310018, China

**Keywords:** quantum image processing, quantum line detection, quantum image transformation

## Abstract

Line detection is a fundamental technique in image processing. It can extract the required information, while the information that does not need attention can be ignored, thus reducing the amount of data. At the same time, line detection is also the basis of image segmentation and plays an important role in this process. In this paper, we implement a quantum algorithm based on a line detection mask for novel enhanced quantum representation (NEQR). We build a quantum algorithm for line detection in different directions and design a quantum circuit for line detection. The detailed module designed is also provided. On a classical computer, we simulate the quantum method, and the simulation results prove the feasibility of the quantum method. By analyzing the complexity of quantum line detection, we find that the computation complexity of the proposed method is improved compared to some similar edge detection algorithms.

## 1. Introduction

Quantum technology has developed rapidly in recent years. Great progress has been made in various fields of quantum information science. In particular, some quantum computers have been developed, such as Google’s quantum hegemony [1], which has brought quantum information research to worldwide attention. Due to the advantages of quantum parallel computation, it has the ability to surpass classical computation in many aspects. For example, the time complexity of quantum algorithms will not grow exponentially as the number of bits increases. Quantum information technology also shows some advantages in image processing; the new research frontier is named quantum image processing (QIP). Quantum information shows its prospects in almost all aspects of image processing. This includes the quantum representation of images for which different representations are already available. In other aspects, quantum versions of image processing have also been developed, including image scrambling [2,3], image scaling [4,5], image transformation [6,7,8,9], image filtering [10,11,12], edge detection [13,14,15,16,17,18,19,20,21,22], image segmentation [23,24], image encryption [25,26,27,28,29,30,31,32,33,34,35,36,37], feature extraction [38], etc. Compared with classical methods, these quantum image processing methods show great advantages. For example, the unique properties of quantum mechanics, such as entanglement, parallelism, and superposition, can be used in image processing, and this can process the pixels simultaneously and provide acceleration in the speed of image processing. The increasing demand for high-quality images today has also led to an increase in image storage space. Quantum image processing can conveniently store larger images and effectively process them in real time.

One class of problems in image segmentation techniques is image line detection. In the process, we are interested in a line in a certain direction, and other lines and other image content can be ignored. After the operation, the information of concern can be obtained and the sizes of image datasets are reduced. Thus, the line detection method is necessary in image processing. In addition, line detection also constitutes the basis of image segmentation, and it has also significant reference value for the construction of image segmentation methods, because image segmentation is always based on lines. Line detection includes horizontal, vertical, +45°, and −45° line detection. In classical image processing, it uses masks to perform convolution or correlation to detect lines. In quantum image processing, using the characteristics of quantum information, the question of how to implement quantum line detection needs to be discussed. Based on some quantum filtering methods [11], such as mean filtering, median filtering, and some edge detection methods [15,19,20], quantum line detection methods are designed, and the quantum circuits also are constructed.

The quantum line detection method is similar to the edge detection method. For edge detection, some works have been completed using the image representation for NEQR—for example, classical Sobel operator detection [15], improved Prewitt operator detection [17], Marr–Hildreth edge detection [18], improved Sobel operator detection [19], eight-direction Sobel operator detection [20], Kirsch operator detection [21], Robinson operator detection [22], etc. Compared with these methods, the proposed line detection method shows some improvements and has its own characteristics. In some edge detection schemes [14,15,21], the segmentation threshold is usually set to be the power of two in order to design the quantum circuit conveniently, while the threshold of our scheme can be set arbitrarily. In addition, the time complexity of our scheme has also been improved in comparison with the above-mentioned methods. Using the same NEQR image representation, our time complexity does not exceed O(*n*^2^ + *q*^2^), but the edge detection algorithm for the classical Sobel operator is no more than O(*n*^2^ + 2*^q^*^+4^) [15], and that of Kirsch operator detection is no more than O(*n*^2^ + 2*^q^*^+3^) [21]. For recent methods reported in [19,22], their time complexity is no more than O(*n*^2^ + *q*^3^) and O(*n*^2^ + 2*^q^*^+3^), respectively. As the number of image bits increases, our method can process images faster. Therefore, our method has advantages.

In this paper, we investigate a line detection method for quantum image processing. The quantum algorithm and quantum circuit for line detection are designed. Using classical digital processing, the quantum line detection for three common test images is simulated with MATLAB on a classical computer. The time complexity of the proposed quantum method is also analyzed. Quantum line detection, which has rarely been studied before, is implemented in our study, which can enrich quantum image processing and provide a reference for other image processing methods.

## 2. The Preparation of Quantum Line Detection

In this section, we describe the preparatory work before implementing quantum line detection, including the implementation method of line detection in classical image processing, the representation method (NEQR) of quantum images used in the paper, and the quantum module used in quantum line detection.

### 2.1. The Classical Line Detection Method

**Figure 1:**
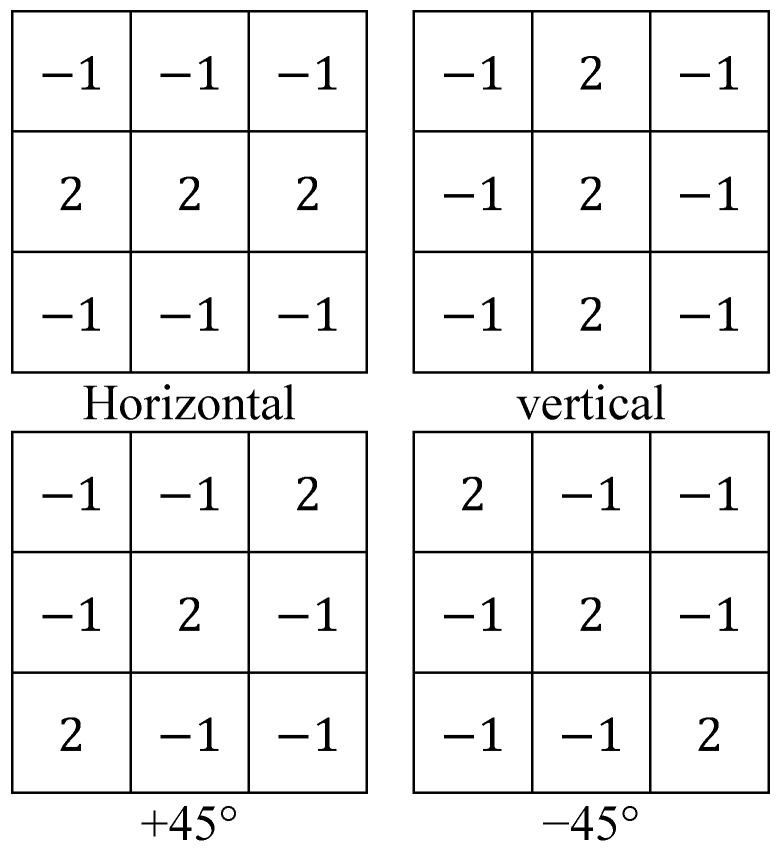
Line detection masks in four directions.

Classical line detection is a method using masks [39]. The process is to apply the line detection mask to the gray-scale image of the original picture. In other words, using masks and the digitized gray-scale image for convolution or correlation calculation, a calculation result can be obtained; then, after setting an appropriate threshold, the new lines detected from the gray-scale image can be obtained. According to the detected lines in different directions, these lines correspond to four types of masks, which are the horizontal mask, vertical mask, +45° mask, and −45° mask [39]. These masks are as follows (Figure 1).

For example, using the horizontal mask, the horizontal line can be calculated by the following equation:

(1) $$L^{H}=\left[\begin{array}{ccc} -1 & -1 & -1\\ 2 & 2 & 2\\ -1 & -1 & -1 \end{array}\right]*p$$

where *p* is a digital image, and *L*^H^ is the detected horizontal line.

### 2.2. Quantum Representation of Image

The quantum representation methods of images are categorized into different types. For example, there are the FRQI [40], MCRQI [41], NEQR [42], GQIR [43], and QIRHSI [44] methods, etc. It is indicated that the NEQR method is more suitable for gray-scale image operation. Since quantum line detection of images involves a large number of gray-scale pixel operations, we use the NEQR representation for line detection in this paper.

The NEQR model stores the gray values and pixel coordinates of an image in a quantum state. For a 2*^n^* × 2*^n^* quantum gray-scale image, its NEQR representation is

(2) $$|I\rangle =\frac{1}{2^{n}}\sum_{Y=0}^{2^{n}-1}\sum_{X=0}^{2^{n}-1}|C_{YX}\rangle |YX\rangle =\frac{1}{2^{n}}\sum_{Y=0}^{2^{n}-1}\sum_{X=0}^{2^{n}-1}|c_{YX}^{q-1}c_{YX}^{q-2}\ldots c_{YX}^{0}\rangle |YX\rangle$$

where $|I\rangle$ is the quantum image, *C_YX_* are gray-scale values, $c_{YX}^{q-1}c_{YX}^{q-2}\ldots c_{YX}^{0}$ are the binary representations of the gray-scale values, and $|YX\rangle$ are pixel coordinates. Thus, $|C_{YX}\rangle \in \{0,1,\ldots ,2^{q}-1\}$, $c_{YX}^{q-1}$, $c_{YX}^{q-2}$,…, $c_{YX}^{0}\in \{0,1\}$. The *q* indicates that there are *q* qubits for binary gray-scale values. For instance, a 2 × 2 gray-scale image can be expressed as

(3) $$\begin{array}{l} |I\rangle =\frac{1}{2}(|0\rangle |00\rangle +|80\rangle |01\rangle +|161\rangle |10\rangle +|255\rangle |11\rangle )\\ \;=\left(\begin{array}{c} |00000000\rangle |00\rangle +|01010000\rangle |01\rangle \\ +|10100001\rangle |10\rangle +|11111111\rangle |11\rangle \end{array}\right) \end{array}$$

**Figure 2:**
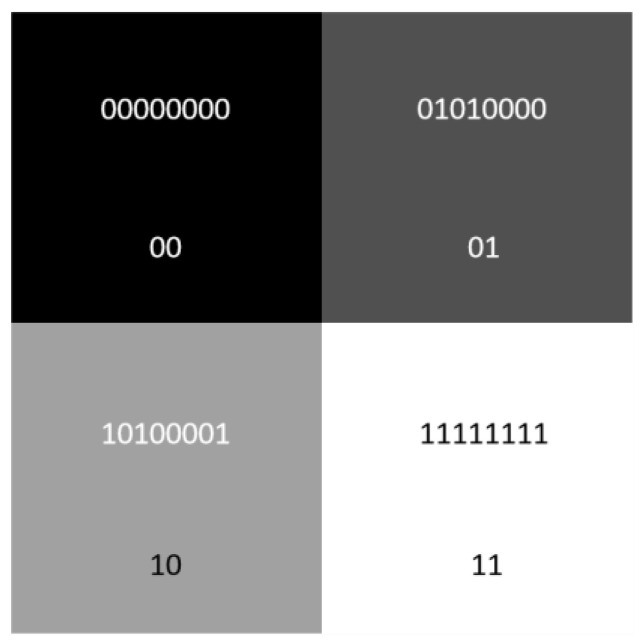
An example of a 2 × 2 image and its NEQR.

The representation expression of the 2 × 2 image in NEQR is shown in Figure 2.

### 2.3. Several Auxiliary Modules for Quantum Line Detection

#### 2.3.1. Quantum Copy Module

**Figure 3:**
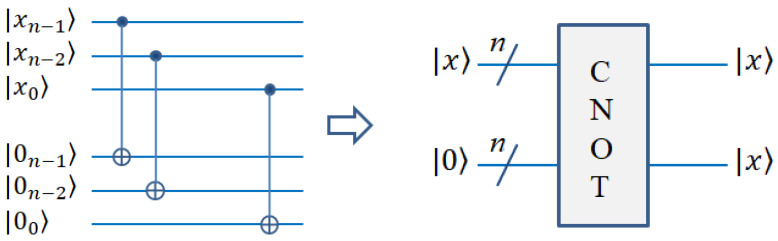
Quantum circuit realization of quantum copy.

Using this module, we can copy a known quantum image. Specifically, given an empty image, the known image is copied to the empty image. The CNOT gate can perform this operation [20]. It is worth noting that the quantum no-cloning principle is not violated here. The quantum no-cloning principle means that the unknown quantum state cannot be copied exactly. Here, the copied quantum state is a known state. The copy module and its simplified diagram are represented in Figure 3.

#### 2.3.2. Quantum Double (DO) Module

**Figure 4:**
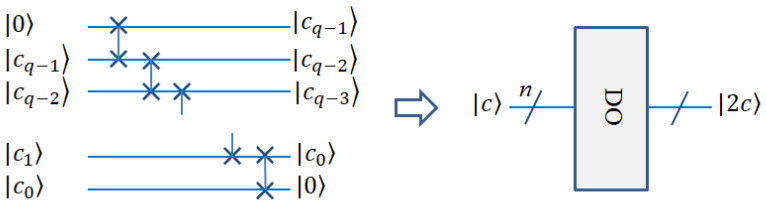
Quantum circuit realization of DO.

In quantum circuit design for line detection, quantum bits need to be multiplied by two. This can be achieved by adding 0 to the last position of the binary bit string. For example, $|c_{q-1}c_{q-2}\ldots c_{0}0\rangle =|2c_{q-1}c_{q-2}\ldots c_{0}\rangle$. The operation can be realized by the auxiliary 0 qubit and the flip gates [15]. The quantum double module is shown in Figure 4.

#### 2.3.3. Cycle Shift Transformation Module

**Figure 5:**
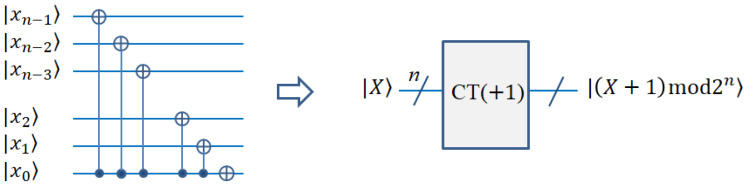
Quantum circuit realization of cycle shift transformation.

The purpose of the cyclic shift transform is to move the whole image. With the transformation operation, the pixel values of the whole image move to the adjacent positions at the same time [19,38]. For example, the right shift operation means that all pixels of the image are shifted to the right by one unit. The right or left and upward or downward shift can be expressed by Equations (4) and (5), respectively. The corresponding circuit diagram is represented and simplified in Figure 5.

(4) $$\mathrm{CT}(X\pm )|I\rangle =\frac{1}{2^{n}}\sum_{Y=0}^{2^{n}-1}\sum_{X=0}^{2^{n}-1}|{\mathrm{CT}}_{YX}\rangle |Y\rangle |X\pm 1\mathrm{mod}2^{n}\rangle$$

(5) $$\mathrm{CT}(Y\pm )|I\rangle =\frac{1}{2^{n}}\sum_{Y=0}^{2^{n}-1}\sum_{X=0}^{2^{n}-1}|{\mathrm{CT}}_{YX}\rangle |Y\pm 1\mathrm{mod}2^{n}\rangle |X\rangle$$

If *X* is used to replace *X* + 1 and *Y* is used to replace *Y* + 1, the above Equations (4) and (5) can be changed to

(6) $$\mathrm{CT}(X\pm )|I\rangle =\frac{1}{2^{n}}\sum_{Y=0}^{2^{n}-1}\sum_{X=0}^{2^{n}-1}|{\mathrm{CT}}_{YX^{\prime }}\rangle |Y\rangle |X\rangle$$

(7) $$\mathrm{CT}(Y\pm )|I\rangle =\frac{1}{2^{n}}\sum_{Y=0}^{2^{n}-1}\sum_{X=0}^{2^{n}-1}|{\mathrm{CT}}_{Y^{\prime }X}\rangle |Y\rangle |X\rangle$$

where $X^{\prime }=(X\mp 1)\mathrm{mod}2^{n}$, and $Y^{\prime }=(Y\mp 1)\mathrm{mod}2^{n}$.

#### 2.3.4. Reversible Parallel Adder (PA) Module

**Figure 6:**
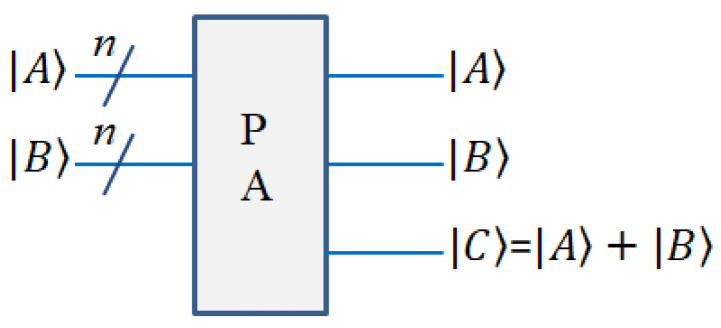
Quantum diagram of PA module.

The reversible parallel adder module is used to calculate the sum of two *n* bit numbers. For example, if $|A\rangle =|a_{n-1}a_{n-2}\ldots a_{1}a_{0}\rangle$, $|B\rangle =|b_{n-1}b_{n-2}\ldots b_{1}b_{0}\rangle$, $|C\rangle =|A\rangle +|B\rangle$ can be calculated through this circuit. This can be achieved through the MAJ (Majority) gate and UMA (Un Majority and Add) gate. In reference [19], more detailed explanations are provided. The circuit diagram is shown in Figure 6.

#### 2.3.5. Quantum Absolute Value Module for Subtraction of Two Positive Integers

To calculate the subtraction of two positive integers, the binary complement is introduced [45]. The subtraction of two positive integers can be regarded as a positive integer plus a negative integer. Here, the binary sequence of integers will become the complement with sign bits. For the representation of the complement, the first bit is the sign bit, where 0 represents a positive number and 1 represents a negative number. The other bits represent the numeric value. For a positive integer, the complement representation is the same as the normal binary representation except for the sign bit. For a negative integer, its numeric bits need to be negated and 1 added. For example, if there are 6 bit representations, for the decimal numbers 5 and −3, their complements are 0 00101, 1 11101, respectively. Generally, for the integer *x* with *n* bits, its complement is

(8) $${\left[x\right]}_{c}=\left\{\begin{array}{c} 0,x_{n-1}x_{n-2}\cdots x_{2}x_{1},\;x_{n}=0\\ 1,{\bar{x}}_{n-1}{\bar{x}}_{n-2}\cdots {\bar{x}}_{2}{\bar{x}}_{1}+1,\;x_{n}=1 \end{array}\right.\;\begin{array}{c} x>0\\ x<0 \end{array}$$

where $|x|=x_{n-1}x_{n-2}\cdots x_{2}x_{1}$, ${\bar{x}}_{l}=1-x_{l}$, *l* = 1, 2, … *n* − 1.

**Figure 7:**
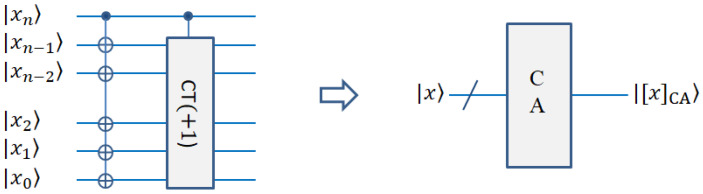

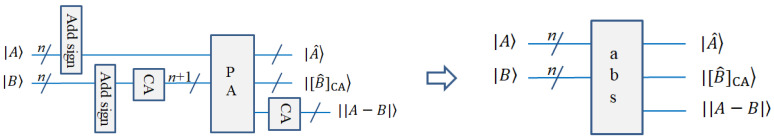
Quantum circuit realization of absolute value. CA module is complement calculation of an integer.

After representation as a complement, the difference between a positive integer and another positive integer can be directly calculated as the complement of the positive integer plus the complement of negative integer. Therefore, the sum of their complements can be directly calculated with the PA module [45]. After this operation, we obtain the result of subtraction of two positive integers. The result is a complement representation with a signed bit. After obtaining the result, we need to calculate the absolute value. As we know, the absolute value is the part of a signed number where the sign bit has been removed. At this time, the number is still represented by a complement and needs to be restored to the original code. There are two cases: if the sign number is 0, it is a positive integer, and its original code is the same as the complement except for the sign bit. In this case, we simply need to remove the sign number to obtain the absolute value. In our complement module (CA) circuit, the sign bit is used as a control bit for complement operation. For positive numbers, the sign bit is 0, so no operation is applied to other bits except for the sign bit. Thus, the positive number will not change after passing through the CA module. As soon as the sign bit is removed, it becomes the absolute value of the positive number. If the sign bit is 1, it is a negative integer. It needs to be changed into the original code, and then the sign bit is removed to obtain the absolute value. The process of changing to the original code is to remove the sign bit firstly, and then invert it (0 convert to 1, 1 convert to 0), and add 1. The process is the same as the operation in which the source code transforms into a complement. For example, for the decimal number −3, its complement is 1 11101, and its original code with a sign bit is 1 00011. In our CA circuit, the sign bit is 1; it is a control bit to perform the conversion operation. At this time, the absolute value can be obtained by removing the sign bit. The circuit diagrams for the complement module and absolute value (abs) module are shown in Figure 7.

#### 2.3.6. Threshold Classification (TC) Module

**Figure 8:**
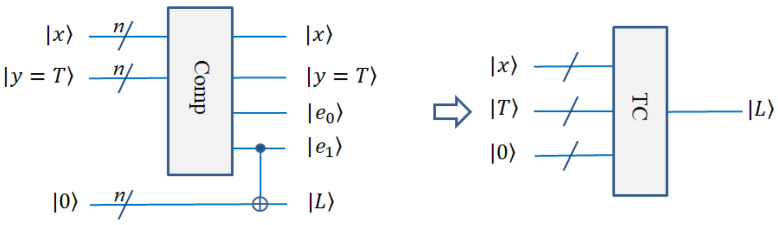
Quantum circuit realization for threshold classification.

In the process of quantum line detection, it is necessary to classify the gray-scale values calculated. This requires us to determine a threshold value. The gray-scale values that are smaller than the threshold value become 0 (black), and those that are larger than the threshold value become 255 (white). This process uses the comparison module, and we use the comparison module in Ref. [18] as part of our circuit. The specific process is as follows. If there are two numbers *x*, *y*, their binary representations are $x=x_{n-1}x_{n-2}\cdots x_{1}x_{0}$, $y=y_{n-1}y_{n-2}\cdots y_{1}y_{0}$, respectively. In a quantum circuit, $|x\rangle$, $|y\rangle$ are inputs, and qubits $e_{1}$ and $e_{0}$ are outputs. If $e_{1}e_{0}$ = 10, then *x* > *y*; if $e_{1}e_{0}$ = 01, then *x* < *y*; and if $e_{1}e_{0}$ = 00, then *x* = *y*. In order to compare it with the threshold value, we take *x* as the gray-scale value and *y* as the threshold value, which can be recorded as $|y=T\rangle$. In order to segment the threshold output, it is necessary to introduce the auxiliary bits, which are ${|0\rangle }^{⊗n}$ bits. Then, the gray-scale values can be classified by the CNOT gate. After this operation, the image is converted into a binary image by threshold value classification. The circuit is shown in Figure 8.

## 3. Quantum Line Detection Algorithm

In this section, we introduce the specific algorithm for the quantum line detection of images. It is based on the modules from the previous section. The algorithm consists of five steps:

(1) Quantum image representation;
(2) Quantum image shift transformation;
(3) Calculation of lines in different directions;
(4) Threshold value classification;
(5) Quantum measurement operation.

### 3.1. Step 1: Quantum Image Representation

As introduced in Section 2.2, we use the NEQR model to represent quantum pictures. For a 2*^n^* × 2*^n^* image, 2*n* position bits are required to represent the pixel position. If the gray-scale range for the image is $0\sim 2^{q-1}$, *q* bits are needed to represent the gray-scale values. For example, if there are 256 gray-scale levels, 8 bits are required to represent the gray-scales.

### 3.2. Step 2: Quantum Image Shift Transformation

**Figure 9:**
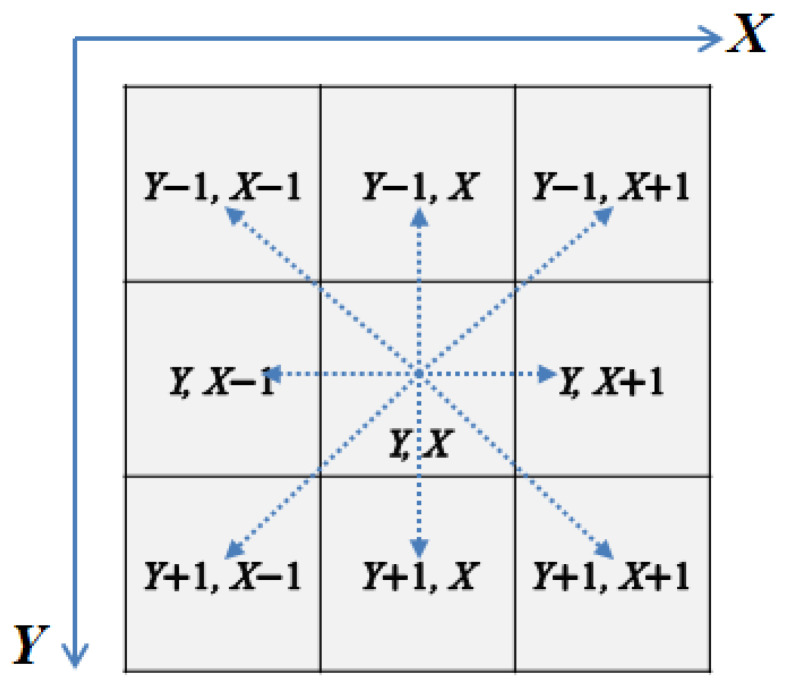
The 3 × 3 neighborhood pixels of pixel (Y, X) and the shift transformation diagram in eight directions.

In classical line detection for an image, the line detection masks (Figure 1) are used to act on the gray-scale image (through convolution or correlation calculation), and desired lines can be detected. However, quantum convolution is very difficult, and even considered unachievable in some works [18]. Here, we first move the original image in eight directions around the pixel coordinate *XY*. By adding moving images to the original image, we can obtain nine images. The gray-scale values in the same position of the nine images are multiplied by the coefficients of the corresponding nine positions in the mask, and the products are added to obtain the same effects, such as classical convolution or correlation between the mask and original image. The moving operators are expressed in Figure 9.

Here, the original image needs to be copied into 8 identical images, which can be done by the quantum copy module.

After the image is copied into eight identical images, we need to use the shift transformation operation to place the eight images at a 3 × 3 vicinity of pixel *XY*. In a quantum circuit, this is done using cycle shift transformation modules. Transformations in eight directions are listed in Algorithm 1. Through the transferring operation, the images become the following quantum state:

(9) $$\begin{array}{l} \frac{1}{2^{n}}\sum_{Y=0}^{2^{n}-1}\sum_{X=0}^{2^{n}-1}|C_{Y-1}{}_{,\;X-1}\rangle ⊗|C_{Y}{}_{,\;X-1}\rangle ⊗|C_{Y+1}{}_{,\;X-1}\rangle ⊗|C_{Y-1}{}_{,\;X}\rangle ⊗|C_{Y}{}_{,\;X}\rangle \\ \;⊗|C_{Y+1}{}_{,\;X}\rangle ⊗|C_{Y-1}{}_{,\;X+1}\rangle ⊗|C_{Y}{}_{,\;X+1}\rangle ⊗|C_{Y-1}{}_{,\;X+1}\rangle ⊗|YX\rangle \end{array}$$

**Algorithm 1** Computation algorithm to shift the image$1.\;\mathrm{Input}:\;\mathrm{the}\;\mathrm{quantum}\;\mathrm{original}\;\mathrm{image}\;I_{YX},\;i.e.,\;|I_{YX}\rangle =\frac{1}{2^{n}}\sum_{Y=0}^{2^{n}-1}\sum_{X=0}^{2^{n}-1}|C_{YX}\rangle |YX\rangle$ $2.\;\mathrm{Shift}\;I_{YX}\;\mathrm{one}\;\mathrm{unit}\;\mathrm{upward},\;\mathrm{then}\;|I_{Y+1X}\rangle =\mathrm{CT}(Y-)|I_{YX}\rangle =\frac{1}{2^{n}}\sum_{Y=0}^{2^{n}-1}\sum_{X=0}^{2^{n}-1}|C_{Y+1X}\rangle |YX\rangle$ $3.\;\mathrm{Shift}\;I_{Y+1X}\;\mathrm{one}\;\mathrm{unit}\;\mathrm{rightwards},\;\mathrm{then}\;|I_{Y+1X-1}\rangle =\mathrm{CT}(X+)|I_{Y+1X}\rangle =\frac{1}{2^{n}}\sum_{Y=0}^{2^{n}-1}\sum_{X=0}^{2^{n}-1}|C_{Y+1X-1}\rangle |YX\rangle$ $4.\;\mathrm{Shift}\;I_{Y+1X-1}\;\mathrm{one}\;\mathrm{unit}\;\mathrm{downwards},\;\mathrm{then}\;|I_{YX-1}\rangle =\mathrm{CT}(Y+)|I_{Y+1X-1}\rangle =\frac{1}{2^{n}}\sum_{Y=0}^{2^{n}-1}\sum_{X=0}^{2^{n}-1}|C_{YX-1}\rangle |YX\rangle$ $5.\;\mathrm{Shift}\;I_{YX-1}\;\mathrm{one}\;\mathrm{unit}\;\mathrm{downwards},\;\mathrm{then}\;|I_{Y-1X-1}\rangle =\mathrm{CT}(Y+)|I_{YX-1}\rangle =\frac{1}{2^{n}}\sum_{Y=0}^{2^{n}-1}\sum_{X=0}^{2^{n}-1}|C_{Y-1X-1}\rangle |YX\rangle$ $6.\;\mathrm{Shift}\;I_{Y-1X-1}\;\mathrm{one}\;\mathrm{unit}\;\mathrm{leftward},\;\mathrm{then}\;|I_{Y-1X}\rangle =\mathrm{CT}(X-)|I_{Y-1X-1}\rangle =\frac{1}{2^{n}}\sum_{Y=0}^{2^{n}-1}\sum_{X=0}^{2^{n}-1}|C_{Y-1X}\rangle |YX\rangle$ $7.\;\mathrm{Shift}\;I_{Y-1X}\;\mathrm{one}\;\mathrm{unit}\;\mathrm{leftward},\;\mathrm{then}\;|I_{Y-1X+1}\rangle =\mathrm{CT}(X-)|I_{Y-1X}\rangle =\frac{1}{2^{n}}\sum_{Y=0}^{2^{n}-1}\sum_{X=0}^{2^{n}-1}|C_{Y-1X+1}\rangle |YX\rangle$ $8.\;\mathrm{Shift}\;I_{Y-1X+1}\;\mathrm{one}\;\mathrm{unit}\;\mathrm{upwards},\;\mathrm{then}\;|I_{YX+1}\rangle =\mathrm{CT}(Y-)|I_{Y-1X+1}\rangle =\frac{1}{2^{n}}\sum_{Y=0}^{2^{n}-1}\sum_{X=0}^{2^{n}-1}|C_{YX+1}\rangle |YX\rangle$ $9.\;\mathrm{Shift}\;I_{YX+1}\;\mathrm{one}\;\mathrm{unit}\;\mathrm{upwards},\;\mathrm{then}\;|I_{Y+1X+1}\rangle =\mathrm{CT}(Y-)|I_{YX+1}\rangle =\frac{1}{2^{n}}\sum_{Y=0}^{2^{n}-1}\sum_{X=0}^{2^{n}-1}|C_{Y+1X+1}\rangle |YX\rangle$ $10.\;\mathrm{Shift}\;I_{Y+1X+1}\;\mathrm{one}\;\mathrm{unit}\;\mathrm{rightwards}\;\mathrm{and}\;\mathrm{one}\;\mathrm{unit}\;\mathrm{downward}\;\mathrm{to}\;\mathrm{the}\;\mathrm{original}\;\mathrm{position},\;\mathrm{then}\;|I_{YX}\rangle =\mathrm{CT}(X+)(\mathrm{CT}(Y+)|I_{Y+1X+1}\rangle )=\frac{1}{2^{n}}\sum_{Y=0}^{2^{n}-1}\sum_{X=0}^{2^{n}-1}|C_{YX}\rangle |YX\rangle$.

### 3.3. Step 3: Quantum Line Calculation in Different Directions

After the transfer operation, the eight gray-scale values adjacent to the *XY* pixel position of the original image will be moved to the *XY* position of the eight new images. Now, by calculating the products of the gray-scale values and the coefficients in the mask, the results, such as convolution or correlation calculation in the classical method, can be obtained. Then, the lines in different directions of the image $|I_{YX}\rangle$ can be calculated using Equations (10)–(13). Lines in four directions are horizontal, vertical, +45°, and −45° lines.

(10) $$\left||L_{YX}^{H}\rangle \right|=\left|\begin{array}{l} \left(|2C_{Y,X-1}\rangle +|2C_{Y,X}\rangle +|2C_{Y,X+1}\rangle )-(|C_{Y+1,X-1}\rangle +|C_{Y+1,X}\rangle +|C_{Y+1,X+1}\rangle \right)\\ -(|C_{Y-1,X-1}\rangle +|C_{Y-1,X}\rangle +|C_{Y-1,X+1}\rangle ) \end{array}\right|$$

(11) $$\left||L_{YX}^{V}\rangle \right|=\left|\begin{array}{l} \left(|2C_{Y-1,X}\rangle +|2C_{Y,X}\rangle +|2C_{Y+1,X}\rangle )-(|C_{Y-1,X+1}\rangle +|C_{Y,X+1}\rangle +|C_{Y+1,X+1}\rangle \right)\\ -(|C_{Y-1,X-1}\rangle +|C_{Y,X-1}\rangle +|C_{Y+1,X-1}\rangle ) \end{array}\right|$$

(12) $$\left||L_{YX}^{+45{}^{\circ}}\rangle \right|=\left|\begin{array}{l} \left(|2C_{Y-1,X+1}\rangle +|2C_{Y,X}\rangle +|2C_{Y+1,X-1}\rangle )-(|C_{Y,X+1}\rangle +|C_{Y+1,X}\rangle +|C_{Y+1,X+1}\rangle \right)\\ -(|C_{Y,X-1}\rangle +|C_{Y-1,X}\rangle +|C_{Y-1X-1}\rangle ) \end{array}\right|$$

(13) $$\left||L_{YX}^{-45{}^{\circ}}\rangle \right|=\left|\begin{array}{l} \left(|2C_{Y-1,X-1}\rangle +|2C_{Y,X}\rangle +|2C_{Y+1,X+1}\rangle )-(|C_{Y-1,X}\rangle +|C_{Y,X+1}\rangle +|C_{Y-1,X+1}\rangle \right)\\ -(|C_{Y+1,X}\rangle +|C_{Y,X-1}\rangle +|C_{Y+1X-1}\rangle ) \end{array}\right|$$

**Figure 10:**
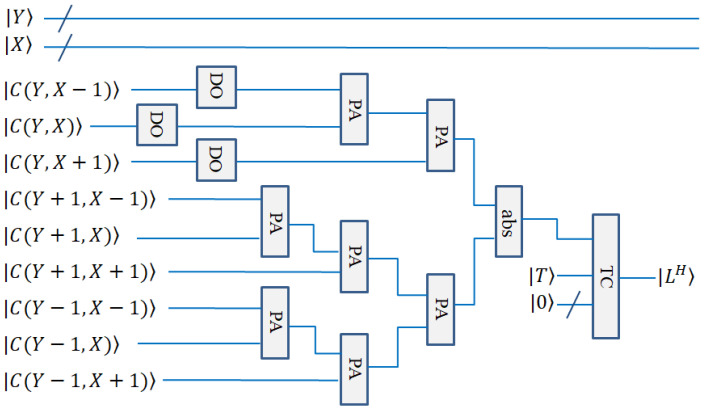
Quantum circuit realization for horizontal line detection.

**Figure 11:**
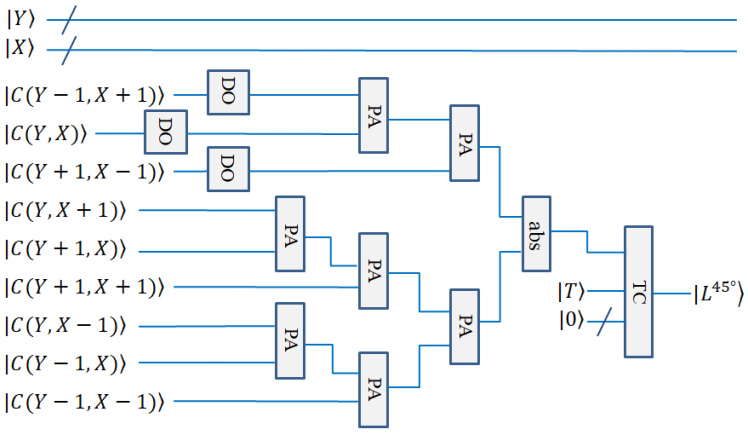
Quantum circuit realization for +45° line detection.

In quantum circuit design, the calculation of the above lines can be performed by the quantum double module, PA module, and quantum absolute value module (the subtraction of converted into complements). The calculation circuits are shown in Figure 10 and Figure 11.

### 3.4. Step 4: Quantum Threshold Value Classification

After calculating the lines in different directions, the obtained images can be classified by the threshold. In other words, the gray-scales below a certain threshold are set to 0, and the gray-scales above the threshold are set to 2*^q^* − 1. It can be expressed by Equation (14). In this way, the image becomes a binary image. The gray-scales that become 2*^q^* − 1 constitute the detected lines. The threshold classification (TC) module can be used to design the quantum circuits.

(14) $$U_{T}\left(\sum_{Y=0}^{2^{n}-1}\sum_{X=0}^{2^{n}-1}|L_{YX}\rangle {|0\rangle }^{⊗2^{q}-1}\right)=\sum_{L_{YX}>T}|2^{q-1}\rangle |YX\rangle +\sum_{L_{YX}\leq T}|0\rangle |YX\rangle$$

where *U_T_* indicates the threshold classification operation.

Through the threshold classification operation, the line detected is represented by a quantum superposition state.

### 3.5. Step 5: Quantum Measurement Operation

After the threshold classification operation, the quantum image needs to be restored to the classical image by quantum measurement. Finally, the quantum line detection is completed. However, line detection is only one step of quantum image preprocessing, and we do not consider the quantum measurement. Its time complexity is also very large (*O*(2^2*n*^)).

The quantum circuit diagrams for the horizontal line detection and +45° line detection are shown in Figure 10 and Figure 11, respectively. The vertical line detection and −45° line detection are not given, because they are similar to the above-mentioned two detections.

## 4. Complexity Analysis

In this section, we analyze the complexity of the quantum line detection circuit. The complexity of the circuit is analyzed according to the gates used in circuits, such as the CNOT gate, Toffoli gate, Hadamard gate, etc. We analyze the complexity based on several steps of line detection.

Firstly, we use the NEQR method to represent images. For a 2*^n^* × 2*^n^* image, there are 2*^q^* − 1 gray-scale levels, and its complexity is no more than O(*qn*2^2*n*^) [42].

Secondly, we perform the cycle shift transformation operation. Before cycle transformation, we need to copy the original image into eight identical copies, and the copy uses the CONT gate. For the CONT gate, its complexity is O(*q*) [15]. For the cycle shift transformation operations, its complexity is O(*n*^2^) [19]. Therefore, the complexity of the whole process is O(*n*^2^ + *q*).

Again, we perform the calculation of lines in different directions. This step uses the quantum double (DO) module, reversible parallel adder (PA) module, and absolute value module (abs). For the quantum double module, its complexity is O(*q*) [20]. The complexity of the reversible parallel adder (PA) is also O(*q*) [19]. However, for the absolute value module (abs), its circuit complexity is O(*q*^2^) [20]. Therefore, the complexity for this step is O(*q*^2^).

Finally, in the threshold classification step, the comparison operation and the CNOT gates are used. For the comparison operation, which is the comparison of the gray-scale values and threshold value, the complexity is O(*q*^2^) [18]. For the CNOT gate, its complexity is O(*q*). In this step, the total complexity is O(*q*^2^ + *q*).

In summary, according to the above analysis, the overall complexity is O[*qn*2^2*n*^ + (*n*^2^ + *q*) + *q*^2^ + (*q*^2^ + *q*)] = O(*qn*2^2*n*^ + *n*^2^ + *q*^2^) in the whole circuit. It is O(*n*^2^ + *q*^2^) except for the NEQR representation. For the completeness of the paper, we give the quantum state representation of the image, line detection processes, and measurement processes for image restoration. However, for general considerations, the NEQR representation for the image and the quantum measurement to restore the image are not considered part of quantum image processing and are not included in the complexity analysis [15,17,19,20]. Thus, for a 2*^n^* × 2*^n^* image, the complexity of quantum line detection processes is O(*n*^2^ + *q*^2^). For the classical line detection process of such an image, its complexity is no less than O(2^2*n*^). The complexity of our method is only a second-order polynomial function, which provides exponential acceleration. The time complexity of our scheme, classical line detection, and some similar edge detection algorithms is listed in Table 1. The first three rows are classic processing methods, and the following rows are the quantum method. It can be seen that our scheme has some advantages compared with other quantum algorithms.

## 5. Simulation Analysis

**Figure 12:**
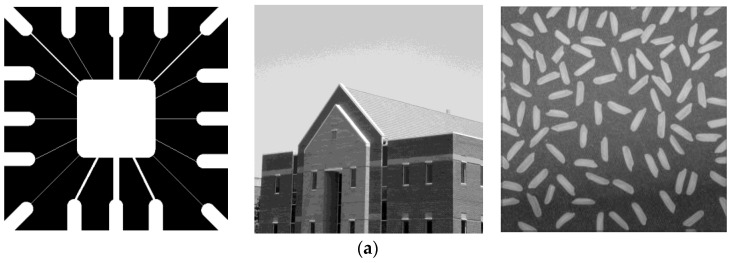

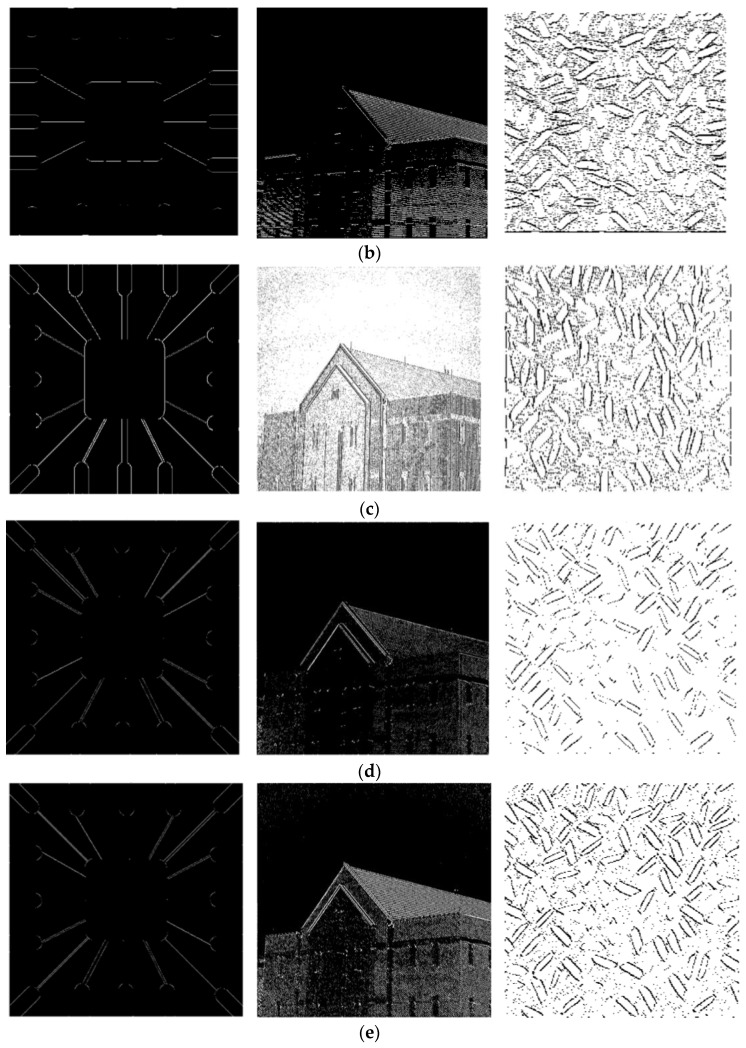
(**a**) Three common and original test images—wiring diagram of circuit board—house—rice. (**b**) The resulting images of quantum horizontal line detection. (**c**) The resulting images of quantum vertical line detection. (**d**) The resulting images of quantum +45° line detection. (**e**) The resulting images of quantum −45° line detection.

In order to test the performance of our quantum algorithm, we simulated the quantum algorithm with MATLAB on a classical computer. MATLAB2014 was used. Our computer simulation used the same steps as the quantum algorithm. It proves the feasibility of the quantum algorithm to some extent. We selected three common test images for the line detection simulation, such as the wiring diagram of a circuit board, a house, and rice (Figure 12a) [39]. In Figure 12b–e, the line detection results for the horizontal, vertical, +45°, and −45° masks are shown, respectively.

For the simulation results, it can be clearly seen that the horizontal and vertical masks mainly detect horizontal and vertical lines, respectively. The +45° and −45° masks are mainly used to detect the lines in the 45° and −45° directions, and oblique lines in other directions can also be detected. The horizontal and vertical masks can partly detect the oblique lines, because the oblique lines are also composed of horizontal and vertical lines. It can be seen from the detection of the wiring diagram of the circuit board, for the +45° mask, that there is more clarity for the diagonal lines close to +45°. In the −45° direction, the lines detected become double lines. The phenomenon is similar for the −45° mask. For the detection of rice, the masks for different directions can detect the corresponding rice closer to the direction. For example, the vertical mask detects more rice in the vertical direction. During the computer simulations, we set the gray-scale threshold to 128 in the wiring diagram and rice detections and 140 for the house detection.

## 6. Conclusions

In this paper, we have developed a quantum line detection method. Based on the NEQR model of representing images, firstly, we introduced the quantum modules for line detection. Based on these quantum modules, we then established the quantum line detection algorithms for the horizontal, vertical, +45°, and −45° directions, and designed the quantum circuit diagram. In a classical computer with MATLAB, we simulated line detections in different directions. The simulation method and the quantum method possess the same steps, and the simulation proves the effectiveness of our quantum method. In the complexity analysis stage, based on the quantum parallel method, we found that the complexity of the proposed scheme is a second-order polynomial function (O(*n*^2^ + *q*^2^)). Compared with other, similar edge detection algorithms, the complexity of our scheme has some advantages. Quantum line detection is computationally faster and it is also the basis for image segmentation. In image processing, line detection can be used in different fields, such as automatic traffic control systems, the location of objects in satellite images, medical imaging, building monitoring, remote sensing monitoring, etc. With the development of quantum technology, quantum line detection algorithms can be directly run in quantum computers to accelerate the speed and increase the storage capacity. Thus, our research has great theoretical and practical implication significance.

## Figures and Tables

**Table 1:** Comparison of the complexity between our scheme and other quantum edge detection algorithms.

Algorithm	QIR Model	Complexity of Quantum Image Construction	Complexity of Algorithm
Line detection	-	-	*O*(2^2*n*^)
Prewitt [17]	-	*-*	*O*(2^2*n*^)
Sobel [19]	-	*-*	*O*(2^2*n*^)
Classical Sobel–Fan [15]	NEQR	*O*(*qn*2^2*n*^)	*O*(*n*^2^ + 2*^q^*^+4^)
Improved Prewitt–Zhou [17]	NEQR	*O*(*qn*2^2*n*^)	*O*(*n*^2^ + 2*^q^*^+3^)
Improved Sober–Chetia [19]	NEQR	*O*(*qn*2^2*n*^)	*O*(*n*^2^ + *q*^3^)
Kirsch–Xu [21]	NEQR	*O*(*qn*2^2*n*^)	*O*(*n*^2^ + 2*^q^*^+3^)
Robinson–Chakraborty [22]	NEQR	*O*(*qn*2^2*n*^)	*O*(*n*^2^ + 2*^q^*^+3^)
Our scheme	NEQR	*O*(*qn*2^2*n*^)	*O*(*n*^2^ + *q*^2^)

## Data Availability

Data underlying the results presented in this paper are not publicly available at this time but may be obtained from the authors upon reasonable request.
